# Phonon limited superconducting correlations in metallic nanograins

**DOI:** 10.1038/srep16515

**Published:** 2015-11-13

**Authors:** M. D. Croitoru, A. A. Shanenko, A. Vagov, M. V. Milošević, V. M. Axt, F. M. Peeters

**Affiliations:** 1 Departement Fysica, Universiteit Antwerpen, B-2020 Antwerpen, Belgium; 2 Universidade Federal de Pernambuco, 50670-901 Recife, Pernambuco, Brazil; 3 Theoretische Physik III, Universität Bayreuth, 95440 Bayreuth, Germany

## Abstract

Conventional superconductivity is inevitably suppressed in ultra-small metallic grains for characteristic sizes smaller than the Anderson limit. Experiments have shown that above the Anderson limit the critical temperature may be either enhanced or reduced when decreasing the particle size, depending on the superconducting material. In addition, there is experimental evidence that whether an enhancement or a reduction is found depends on the strength of the electron-phonon interaction in the bulk. We reveal how the strength of the *e-ph* interaction interplays with the quantum-size effect and theoretically obtain the critical temperature of the superconducting nanograins in excellent agreement with experimental data. We demonstrate that strong *e-ph* scattering smears the peak structure in the electronic density-of-states of a metallic grain and enhances the electron mass, and thereby limits the highest *T*_*c*_ achievable by quantum confinement.

A recent experiment by Bose *et al.*^1^ revealed material-dependent quantum size effects in superconducting grains which led to reconsider an old but fundamental problem of superconductivity in confined systems. Over the last 50 years the properties of nanoscale superconductors have been investigated extensively, both theoretically and experimentally, due to their general importance for the understanding of the nature of the ground state in confined systems^2^. The question of how the superconducting properties of confined systems are altered with thickness down to the nanometer size range has also significant technological ramifications. Anderson^3^ argued that conventional superconductivity should disappear for sample sizes such that the average level spacing *δ* (inversely proportional to the volume of the grain) is of the order of the BCS gap Δ. Blatt and Thompson in their pioneering work predicted that above the Anderson limit the critical temperature *T*_*c*_ exhibits quantum oscillations with remarkable thickness-dependent resonant enhancements^4^. Subsequent experimental studies of superconducting confined systems showed either a decrease or an increase of *T*_*c*_ with sample size, depending on the material.

Since 1960s most of the experiments devoted to superconducting correlations in grains were performed with either grain powders or with granular films, where each metallic crystallized grain is surrounded by an insulating, amorphous coating (barrier)^5^,^6^,^7^,^8^,^9^,^10^,^11^,^12^,^13^,^14^,^15^,^16^,^17^,^18^,^19^. The experimental results are summarized in Table 1. The increase of *T*_*c*_ observed for In, Sn, and Al was attributed either to quantum-size effects^20^,^21^,^22^,^23^,^24^,^25^,^26^,^27^,^28^,^29^,^30^,^31^,^32^,^33^,^34^,^35^,^36^ or to the softening of the phonon modes due to the presence of the surface^37^,^38^,^39^,^40^,^41^,^42^. The studies showed good agreement between theory and experiment for weak- and intermediate-coupling superconductors. However, the same theoretical prescriptions failed to show any observable size effects on *T*_*c*_ for the strong-coupling material Pb. Studies of nanostructured Pb and Nb films consisting of crystalline grains separated by a disorder inter-granular region, making the system similar to a disordered network of weakly coupled grains, revealed a decrease of *T*_*c*_ with decreasing thickness^14^,^16^.

A scanning tunneling microscope (STM) was used to detect the superconducting gap in a single physically isolated ultra-small Pb/Sn grain^1^,^43^,^44^. These studies traced the size evolution of superconductivity in isolated nanoparticles that were grown on a substrate. It was demonstrated that, while in both systems (Pb and Sn) superconductivity is ultimately quenched at the Anderson limit, the size dependence of *T*_*c*_ before the destruction of superconductivity was different. In a Sn particle oscillations of the superconducting energy gap with particle size were observed and the enhancement of the gap was reported to be as large as 60%. Contrarily, Pb particles exhibited a decrease of the gap with decreasing particle size. The theoretical explanation was based on a model of the density-of-states (DOS), which included a phenomenological broadening parameter due to scattering or recombination processes, escape rates, and instrumental broadening. A recent study was devoted to the role of the substrate in the decay of *T*_*c*_ with sample size^45^.

Recently, a series of experimental results obtained with samples made of superconducting Pb islands with thickness between 5 and 60 monolayers and grown on Si(111) substrate were reported^46^,^47^. These superconducting samples can be considered as quasi-2D superconducting islands with strong effects of level quantization. A reduction of the superconducting gap of ultrathin islands was observed. Layer-dependent *ab initio* density functional calculations for freestanding Pb films showed that the electron-phonon coupling decreases with decreasing film thickness and hence results in the size-dependent destruction of superconductivity^44^. Interestingly the suppression of superconductivity with size depends to a good approximation only on the volume of the island and is independent of its shape^47^.

Therefore, the above overview of the experimental findings over the last 50 years shows that the behavior of *T*_*c*_ changes from an increase to a decrease with sample size reduction as the strength of the *e-ph* interaction increases. In this article we show that the experimental results can be explained by an interplay of quantum confinement for the electronic degrees of freedom and a phonon environment. The key ingredients of the mechanism is the inelastic electron phonon scattering that is sufficiently strong to smear the discrete energy spectrum of the particle into a continuum^48^ and the mass renormalization due to the electron-phonon scattering. Thus, in addition to the *pair-forming effect* due to virtual phonons inherent to the BCS model we take into account also the *pair-breaking effect* due to thermal phonons inevitably present in any solid-state system. Since the quantum-size effect is inversely proportional to the electron mass it is seen that the effect is due to the *e-ph* mass-enhancement parameter *λ*, defined as the frequency derivative of the electron self-energy due to the *e-ph* interaction.

## Results

### The theory behind the model

The electronic states in metal structures are subject to different scattering mechanisms. While the influence of imperfections (impurities, surface roughness, layer-width fluctuations) can, at least in principle, be controlled by improved technology, phonon scattering is inherent to the solid state of matter. The general Hamiltonian for a coupled electron-phonon system interacting via a linear interaction is given by


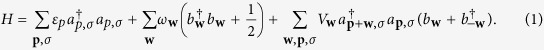


Here *p* is integer numbering the single-particle energy levels *ε*_*p*_, the operator *a*_*p*,*σ*_ 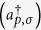 annihilates (creates) an electron in state *p* with spin *σ* and the operator *b*_**w**_ 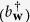 annihilates (creates) a phonon in state **w**. We describe the phonons by a Debye spectrum, i.e., we use 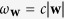, where *c* denotes the velocity of sound and *w* ranges from zero to *w*_*D*_, the Debye wave number. The corresponding maximum phonon energy is denoted by *ω*_*D*_. Here coupling *V*_**w**_ is related with the e-ph coupling used in superconductivity as


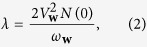


where 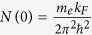. Here *k*_*F*_ denotes the Fermi wave vector. For our quantitative studies devoted to the explanation of the material and size dependence of the superconducting critical temperature Δ*T*_*c*_ we have used bulk phonon parameters, thus neglecting any change of the phonon spectrum due to the presence of the surface. This approach is motivated by the large ratio of electron and phonon quantum-confinement energies which scale as *m*_*ion*_/*m*_*e*_. Nevertheless, the influence of the size-dependent coupling constant on the superconducting characteristics can be essential for ultra-small grains and this issue was studied in ref. 43. The phonon induced contribution to the level broadening can be expressed in terms of the spectral Eliashberg function *α*^2^*F*(*ω*)^49^,^50^


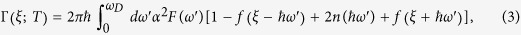


where *f*(*ξ*) and *n*(*ħω*′) are the electron and phonon occupation numbers, respectively. With increasing sample size the energy separation between different single-electron levels decreases and more levels move into the Debye window, thereby opening additional inter-level scattering channels. Therefore, the use of 3D acoustic (LA) *e-ph* scattering should be adequate for calculations of the acoustic phonon-induced single-electron level broadening when the average energy spacing 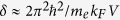 is sufficiently smaller than the mean LA phonon energies. Here *V* is the sample volume. Within the 3D Debye model the Eliashberg function is 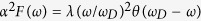
51,52, which results at *T* = 0 in the level broadening:


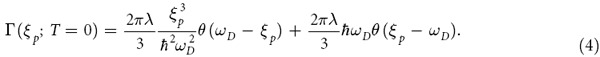


At the Fermi level *ξ* = 0 and finite *T* we obtain^53^:


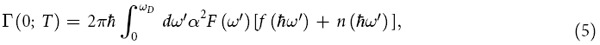


which results into 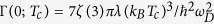. This model requires one external parameter, i.e., the mass-enhancement parameter *λ*.

To describe the superconducting state we consider the reduced Bardeen-Cooper-Schrieffer (BCS) pairing Hamiltonian, where only the time-reversal states are paired


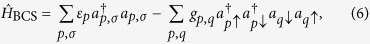


where the interaction matrix element *g*_*qp*_ is given by


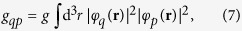


with *g* denoting the coupling constant and *φ*_*q*_(**r**) the single-electron wave function. The first term in Eq. (6) contains the single- electron energies, and the second term is the attractive (when *g* > 0) pairing interaction due to the exchange of virtual phonons. We have assumed that the electron-electron interaction is unaffected by quantum confinement and it is the same as in the bulk. In the bulk the real inter-electron potential is well approximated by a *δ*-function pseudopotential. Employing such a simplified interaction requires a regularization, which makes the matrix elements non-zero only between states within the Debye window around the Fermi surface.

As shown in the Methods section the pairing gap equation is


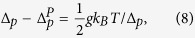


with


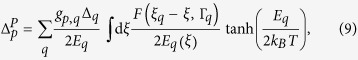


where *E*_*p*_ is the quasi-particle energy given by 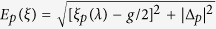 within the pairing interval (Debye window) and 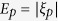 outside. Here 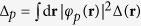, 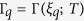 is the total width of the *q*-level, which is approximated by the phonon contribution given in Eq. (3), and the single electron spectrum is described by 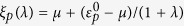 with 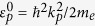. This expression takes into account the modification of the single-electron spectrum due to the electron-phonon interaction, tied to a thin energy shell of approximate width 2*ħω*_*D*_. The function 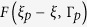 describes the shape of the broadened levels. Since we are dealing with a system at very low temperature the spectrum of a broadened level is asymmetric around *ξ*_*p*_. However, for the purpose of our work this is an unnecessary detail and we assume a symmetric spectral line^54^. If one also assumes that 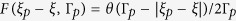, where *θ*(*x*) is the Heaviside step function, then the integral can be done analytically (for *T* → 0)^55^ and we obtain


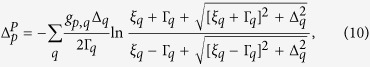


The solution of Eq. (8) gives


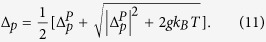


From the pairing gap one can find the density gap 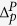. This expression is used in our simulations. It accounts for the phonon-induced enhancement of the level density in the quasi-particle spectrum by a factor 1 + *λ*.

### Numerical results

**Figure 1:**
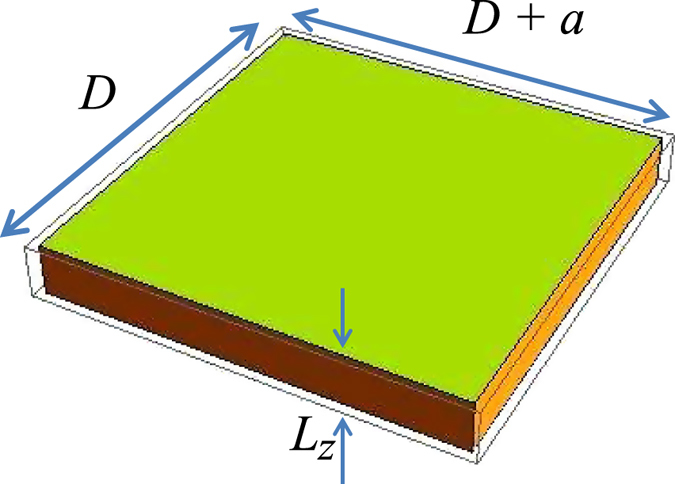
Scheme of the superconducting nanograin.

We model grains as nanoparticles with *L*_*x*_ = *D*, *L*_*y*_ = *D* + *a* and *L*_*z*_ = 2 nm (see Fig. 1), where we vary the lateral size *D* and take *a* = 0.05 nm (a finite *a* is added to avoid the strong degeneracy of the single-electron levels typical for highly-symmetric samples). In our numerics we include the level broadening due to the electron-phonon interaction. The calculations were performed with parameters typical for aluminium, tin, niobium, and lead (see Table 2).

**Figure 2:**
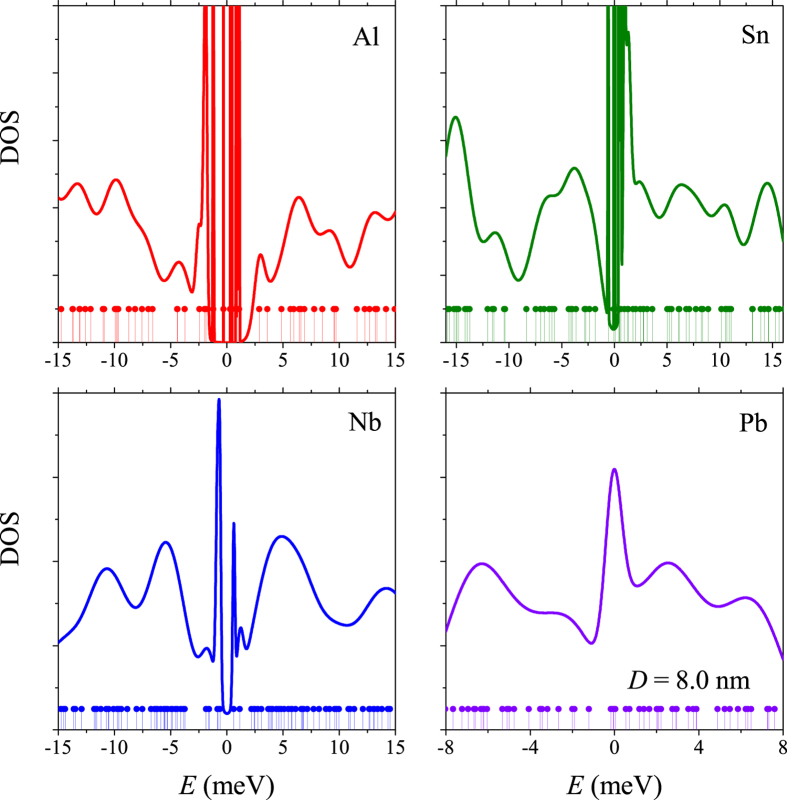
Energy dependence of the DOS for metallic aluminium, tin, niobium, and lead grains with *D* = 8 nm. The dots at the bottom of each figure show the position of the unbroadened single electron levels taken as delta functions.

Figure 2 shows the calculated DOS,


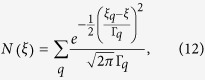


in the Debye window around the Fermi level for aluminium, tin, niobium and lead nanoparticles. The dots at the bottom of the figures show the position of the unbroadened single electron levels. For aluminium and tin particles the behavior of *N*(*ξ*) is as expected: near the Fermi level it remains a collection of separate narrow peaks, reminiscent of the broadening function, centered at the single electron energies *ξ*_*n*_. Evidently, the discreteness of the original spectrum is strongly suppressed for the higher excited electronic states due to the broadening. For niobium and lead nanoparticles the phonon-induced broadening of the peaks is stronger due to the higher *T*_*c*_ and the smaller *ω*_*D*_ (for lead). The result is a rather featureless DOS, which decreases with increasing energy, eventually approaching the average DOS. When increasing the sample size, the DOS approaches a constant typical for bulk materials.

**Figure 3:**
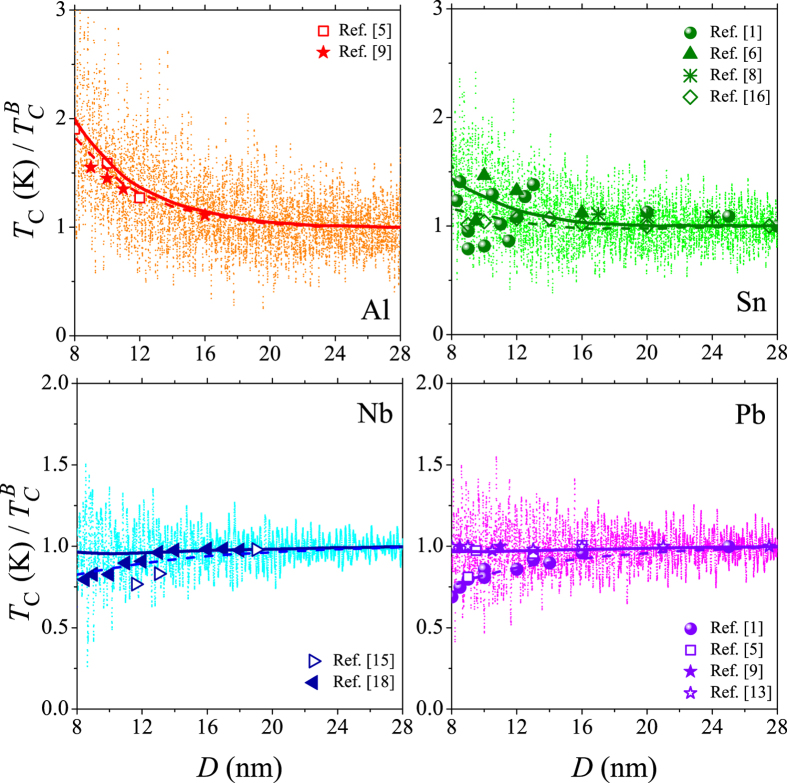
Calculated variation of the critical temperature with the sample size *D* for aluminium, tin, niobium, and lead nanograins. The solid curves give the average dependence. The large symbols show the experimental results. The dashed lines show *T*_*c*_ in disordered samples [see Eq. (13)].

**Figure 4:**
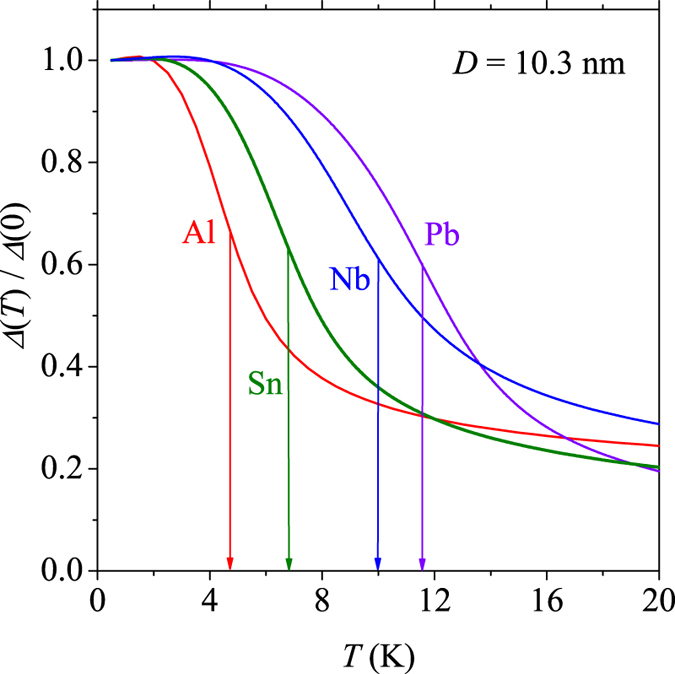
The averaged gaps for Al, Nb, Sn and Pb samples calculated within the reduced SPA formalism. The arrows show the critical temperature extracted at the steepest descent of the superconducting order parameter.

Figure 3 illustrates our results for the normalized reduced critical temperature, 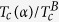, as a function of the sample size for aluminium, tin, niobium and lead metallic nanoparticles. Here 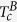 is the superconducting onset temperature for a sample with *D* → ∞. For each sample size the transition temperature, *T*_*c*_, was defined as the temperature which corresponds to steepest descent of the superconducting order parameter as shown in Fig. (4), which displays the values of Δ_*P*_, calculated according to Eq. (9) for a grain with *D* = 10.3 nm, as an example. The curves of the plot are the results for aluminium, tin, niobium and lead. The vertical lines indicate the temperatures at which the decay of the corresponding gaps are the strongest.

The order parameter takes into account thermal fluctuations on the level of reduced static-path approximation (SPA) or effective BCS. All our numerical results exhibit a feature typical for the size-dependent pairing in high-quality superconducting particles: *T*_*c*_ fluctuates with sample size. The amplitude of the fluctuations increases with decreasing *e-ph* coupling. Qualitatively, the fluctuations of *T*_*c*_ can be understood as follows. The pair correlations are non-zero only within a finite range marked by the Debye window around the chemical potential, *μ*. Moreover, the main contributions to the sum in Eq. (10) come from states in the vicinity of the Fermi level. When varying the size of the grain, the number of states in the Debye window changes. The smaller the grain, the smaller the number of relevant states contributing to the pair correlations. However, because of the enhanced broadening of the single-electron levels in materials with stronger *e-ph* coupling, the *T*_*c*_ fluctuations are weaker in these nanoparticles.

The solid curves in Fig. 3 are a guide for the eye and give the average size dependence of *T*_*c*_ in the range of grain widths *D* = 8 − 28 nm. For aluminium and tin nanoparticles we observe an overall increase of *T*_*c*_ by a factor of 2 in the case of aluminium and by a factor of 1.4 in the case of tin when decreasing *D* from bulk to *D* ≈ 8 nm. In contrast, the size dependencies of *T*_*c*_ are almost absent for niobium nanoparticles down to *D* ≈ 8 nm. The transition temperature shows a slight decrease of 4% when the width of the sample is decreased from *D* ≈ 30 nm to *D* ≈ 8 nm. A further reduction of the particle size leads to a slight increase of *T*_*c*_ before its suppression. Lead nanoparticles exhibit a small decrease of *T*_*c*_ with decreasing sample size. The transition temperature decreases by 3–4% when the width of the sample is decreased from *D* ≈ 30 nm to *D* ≈ 8 nm. The different behavior of Nb and Pb nanoparticles as compared with Al and Sn is a direct consequence of the fact that quantum confinement is smaller due to (*i*) the heavier electron mass and (*ii*) larger broadening of the single electron levels, both as a result of the stronger electron-phonon interaction.

## Discussion

We presented a theoretical explanation for the puzzling very different dependence of *T*_*c*_ on grain size in different superconductors. To realize this we presented a theory for the superconducting correlations in nanograins coupled to a phonon environment. We showed how environmental entanglement emerges in the ground-state of these systems and why it has a strong influence on the superconducting characteristics. The *pair-breaking effect* due to thermal phonons was accounted for, which broadens the single electron levels, as well as the virtual phonons that renormalize the band electron mass. Without such level broadening and mass renormalization *T*_*c*_ always increases with decreasing sample size above the Anderson limit. In our theory, as corroborated by experimental observations, quantum confinement can either increase or show constant behaviour/slight decrease of the average superconducting critical temperature, depending on the material parameters. Our analysis conclusively shows that a slight decrease of *T*_*c*_ for smaller samples is expected for the strongly phonon-coupled nanoparticles of lead while an increase is typical for samples made of weakly phonon coupled superconducting materials, both in accordance with experimental findings.

In the above study, we have not accounted for the influence of disorder^56^. We note that in the presence of disorder the level broadening can be size-dependent, because the effect of disorder typically increases with the reduction of sample size due to e.g. surface-roughness, resulting in a stronger level broadening at small sizes. As shown in ref. 57, disorder also results in a repulsion of the energy levels in small metallic samples making them evenly distributed, which additionally decreases *T*_*c*_ when reducing the sample size. As was found by Altshuler and Aronov the Coulomb interaction combined with impurity scattering produces a dip in the density-of-states at the Fermi level, that in turn further decrease the transition temperature with decreasing sample size. To estimate the effect of disorder we use a weak disorder model for homogeneous superconducting samples established by Finkelstein^58^, according to which the suppression of superconductivity is driven by impurities that reinforce Coulomb and spin interactions. According to this model the critical temperature is found from the following expression


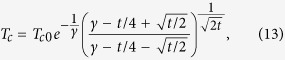


where *T*_*c*0_ is the critical temperature in the absence of disorder, 
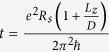
, 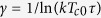 and the diffusion time we define as *τ* = *D*/*v*_*F*_, where *v*_*F*_ is the Fermi velocity. Here *R*_*s*_ is the sheet resistance, *τ*_0_ is the diffusion time for a sample with large lateral size. Within this model we obtain the results shown as dashed lines in Fig. 3. For the calculations we adopted the following parameters *R*_*s*_(Al) = 14.1Ω, *R*_*s*_(Sn) = 54Ω, *R*_*s*_(Pb) = 110Ω, *R*_*s*_(Nb) = 90Ω.

## Methods

In the case of finite superconducting systems the mean field approximation exhibits several drawbacks. The most evident is the presence of sharp phase transitions, which is characteristic of very large systems. This effect is due to statistical fluctuations. The static-path approximation (SPA) provides a microscopic way to include the static fluctuations of the mean field. SPA considers only the static paths in the path integral representation of the partition function. In this paper we have used the reduced SPA formalism within which the statistical sum is written as^59^,^60^


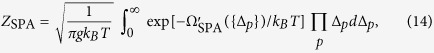


with


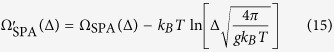


and


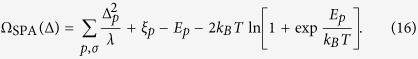


The minimum of 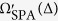 results in Eqs. (8, 9). From the pairing gap one can find the density gap 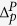.

## Additional Information

**How to cite this article**: Croitoru, M. D. *et al.* Phonon limited superconducting correlations in metallic nanograins. *Sci. Rep.* **5**, 16515; doi: 10.1038/srep16515 (2015).

## Figures and Tables

**Table 1:** Experimental evidence of the critical temperature variation with the grain thickness.

Metal	*d* (nm)	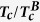	*d* (nm)	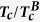	*d* (nm)	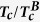
Al	10	1.45^9^	5	2.16^7^	4	2.60^5^
Sn	13	1.14^8^	11	1.10^5^	7	1.14^6^
In	11	1.10^5^	10	1.15^9^	10	1.05^13^
Nb	17	0.97^17^	11	0.62^14^	5	0.74^18^
Pb	11	1.00^5^	10	1.00^9^	6	0.93^19^

**Table 2:** Material parameters^61^.

Metal	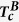 (K)	*ω*_*D*_ (K)	*n*_*e*_ (nm^−3^)	1 + *λ*
Pb	7.19	105	132	2.55
Nb	9.20	275	56	2.84
Sn	3.75	200	148	1.72
Al	1.19	428	180	1.43
